# Methodological considerations in the multicenter cohort study of partial-thickness supraspinatus tear repair by Audigé et al.

**DOI:** 10.1186/s13018-026-07124-4

**Published:** 2026-07-18

**Authors:** Ali Can Koluman, Nezih Ziroglu, Kerem Bilsel

**Affiliations:** 1 Department of Orthopaedics and Traumatology, Bakirkoy Dr. Sadi Konuk Training and Research Hospital, 34147 Istanbul, Turkey; 2 Department of Orthopedic Prosthetics and Orthotics, Vocational School of Health Services, Acibadem Mehmet Ali Aydinlar University, 34638 Istanbul, Turkey; 3 Department of Orthopaedics and Traumatology, Acibadem University Atakent Hospital, 34303 Istanbul, Turkey; 4 Department of Orthopaedics and Traumatology, Acibadem University Fulya Hospital, 34349 Istanbul, Turkey

**Keywords:** Partial-thickness rotator cuff tear, Arthroscopic repair, Tear completion, Postoperative stiffness, Ellman classification, Multicenter cohort

## Abstract

This correspondence comments on the multicenter cohort study by Audigé et al. (Journal of Orthopaedic Surgery and Research, 2026) reporting outcomes of arthroscopic repair for partial-thickness supraspinatus tears. We highlight four aspects of the study that, in our view, would benefit from further clarification or additional discussion: (1) limited information regarding the surgical indications for tear completion, which may complicate interpretation of treatment-selection effects; (2) the absence of a technique-specific analysis of adverse events, particularly postoperative stiffness; (3) the unreported methodology used for intraoperative Ellman grade determination, the primary classification variable and the only factor significantly associated with tear completion; and (4) the potential residual influence of differential concomitant acromioplasty across tear-location groups despite statistical adjustment. Addressing these issues would further strengthen the interpretation and clinical applicability of this otherwise valuable prospective multicenter dataset.

## Introduction

We read with great interest the recent article by Audigé et al. reporting prospective multicenter outcomes of arthroscopic repair for partial-thickness supraspinatus tears (PTRCTs) from 19 Swiss and German centers [1]. Partial-thickness rotator cuff tears remain a heterogeneous clinical entity with evolving treatment strategies and ongoing controversy regarding optimal management [2–6]. The authors are to be commended for presenting one of the most comprehensive PTRCT datasets to date, with standardized adverse event documentation and 24-month follow-up. We wish to highlight four aspects of the study that we believe warrant further clarification or discussion to facilitate interpretation of the reported findings.

## Limited information regarding indications for tear completion

The study reports that 71% of supraspinatus PTRCTs involved more than 50% tendon thickness, whereas tear completion was performed in only 46% of articular-sided and 35% of bursal-sided tears. As expected in a prospective observational cohort reflecting routine clinical practice, surgical decision-making was left to the discretion of the treating surgeons rather than being guided by a predefined protocol. Consequently, the cohort encompasses a broad spectrum of operative strategies. Additional information regarding the indications that led surgeons to perform tear completion—even if obtained retrospectively from operative records—would substantially improve interpretation of the reported findings.

Contemporary evidence continues to emphasize tear depth as the principal factor guiding treatment selection, with structured comparisons primarily focusing on transtendon repair versus tear-completion repair for high-grade lesions [2–4]. Although variability in surgical decision-making is an inherent feature of observational cohorts, retrospective clarification of the indications for tear completion would add clinically relevant information beyond the descriptive reporting of routine practice and improve interpretation of the reported findings. Without a clearer description of the factors influencing treatment selection in the present cohort, it is difficult to determine to what extent the reported outcomes reflect differences in tear location, surgical decision-making, or both.

## Absence of technique-specific analysis of postoperative stiffness and adverse events

The study reports an overall postoperative stiffness rate of 13.2% and presents adverse events stratified by tear location, but not by repair technique. Multiple meta-analyses comparing in situ/transtendon repair with tear-completion repair have reported no significant differences in functional outcomes or overall complication rates [7, 8]. However, technique-specific data on postoperative stiffness remain limited, with a pooled incidence of approximately 10.3% following arthroscopic repair of partial-thickness rotator cuff tears [9]. Given that this prospective multicenter cohort included the major contemporary repair strategies, it provides a valuable opportunity to further characterize postoperative stiffness and other adverse events according to repair technique. We acknowledge that some subgroup analyses, particularly for intratendinous tears, may be underpowered because of the limited number of patients. Nevertheless, reporting these data, even as exploratory analyses, could provide clinically useful information and help inform future comparative studies.

## Clarification of the methodology for Ellman grade determination

Tears were classified intraoperatively according to the Ellman grading system [10], yet the manuscript does not describe the method used to determine tear depth. This is particularly relevant because Ellman grade was the only factor significantly associated with the decision to perform tear completion (*p* = 0.001), meaning that any systematic error in grading could directly influence the primary analysis.

The Ellman classification is based on tear depth thresholds (< 3 mm, 3–6 mm, and > 6 mm), making accurate intraoperative assessment essential for appropriate classification. Because it relies on millimeter-based thresholds, a brief description of how tear depth was measured intraoperatively (e.g., with a calibrated probe) would improve reproducibility, strengthen confidence in the classification process, and facilitate interpretation of the reported findings.

## Potential residual influence of differential concomitant acromioplasty

Acromioplasty was performed substantially more often in bursal-sided (68%) and intratendinous (70%) tears than in articular-sided tears (46%). A recent systematic review and meta-analysis identified concomitant subacromial decompression as a significant risk factor for postoperative shoulder stiffness after rotator cuff repair (odds ratio, 3.26; 95% confidence interval, 1.88–5.65) [11]. Because postoperative range of motion and stiffness were among the principal outcomes compared across tear-location groups, differential use of concomitant acromioplasty may therefore have contributed to the observed differences in addition to tear location itself.

Although the authors appropriately adjusted for acromioplasty in their mixed-effects models and acknowledged the possibility of residual confounding in the limitations section, we believe that the potential residual influence of differential acromioplasty across tear-location groups deserves more explicit discussion. Such clarification would assist readers in determining whether the reported differences primarily reflect tear location or, at least in part, differences in the accompanying surgical procedures.

These observations are intended to complement, rather than diminish, the contribution of this valuable multicenter cohort. We believe that additional clarification regarding treatment selection, intraoperative tear grading, technique-specific adverse events, and the potential influence of concomitant procedures would further strengthen the interpretation of the findings and enhance the clinical value of this important prospective multicenter cohort.

## Data Availability

No datasets were generated or analysed during the current study.
